# Ultrastable Surface‐Dominated Pseudocapacitive Potassium Storage Enabled by Edge‐Enriched N‐Doped Porous Carbon Nanosheets

**DOI:** 10.1002/anie.202005118

**Published:** 2020-06-08

**Authors:** Fei Xu, Yixuan Zhai, En Zhang, Qianhui Liu, Guangshen Jiang, Xiaosa Xu, Yuqian Qiu, Xiaoming Liu, Hongqiang Wang, Stefan Kaskel

**Affiliations:** ^1^ State Key Laboratory of Solidification Processing Center for Nano Energy Materials School of Materials Science and Engineering Northwestern Polytechnical University and Shaanxi Joint Laboratory of Graphene (NPU) Xi'an 710072 P. R. China; ^2^ Inorganic Chemistry Technische Universität Dresden Bergstrasse 66 01062 Dresden Germany; ^3^ State Key Laboratory for Supramolecular Structure and Materials College of Chemistry Jilin University Changchun P. R. China

**Keywords:** nitrogen doping, porous carbon, potassium storage, ultrastable cycling

## Abstract

The development of ultrastable carbon materials for potassium storage poses key limitations caused by the huge volume variation and sluggish kinetics. Nitrogen‐enriched porous carbons have recently emerged as promising candidates for this application; however, rational control over nitrogen doping is needed to further suppress the long‐term capacity fading. Here we propose a strategy based on pyrolysis–etching of a pyridine‐coordinated polymer for deliberate manipulation of edge‐nitrogen doping and specific spatial distribution in amorphous high‐surface‐area carbons; the obtained material shows an edge‐nitrogen content of up to 9.34 at %, richer N distribution inside the material, and high surface area of 616 m^2^ g^−1^ under a cost‐effective low‐temperature carbonization. The optimized carbon delivers unprecedented K‐storage stability over 6000 cycles with negligible capacity decay (252 mA h g^−1^ after 4 months at 1 A g^−1^), rarely reported for potassium storage.

## Graphical abstract

**Pyrolysis–etching** of a pyridine‐coordinated polymer was used to prepare a porous carbon material with defect edge‐enriched N‐6/N‐5 doping, high surface area, and rich distribution of N defects within the material. This carbonaceous material allows full utilization of surface‐dominated capacitive K storage with ultrastable cycling.

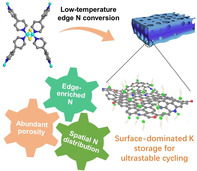

## Introduction

Limited Li resources in the earth's crust (20 ppm) and the ever‐increasing demands for large‐scale electrical energy storage have stimulated numerous efforts for alternative energy storage systems beyond the state‐of‐the‐art Li‐ion batteries.^1^ K‐ion based energy storage systems, such as K‐ion batteries or related hybrid capacitors, have recently emerged as promising candidates based on abundant K resources (17 000 ppm), low cost, and relatively low redox potential (−2.93 V vs. standard hydrogen electrode (SHE)) compared to Li (−3.04 V vs. SHE).1b, 2 A key requirement to realize high performances for K‐ion based energy storage lies in developing stable anode materials, as huge anode volume variation and sluggish kinetics are typically associated with large‐sized K ions.2c, 2d Thus, recent years have witnessed an explosive growth of exploration. Various alloying and intercalating anodes, such as metals,^3^ transition‐metal selenides/sulfides,^4^ and carbonaceous materials have been proposed.2d, 5 Carbonaceous materials have been demonstrated to be among the most promising candidates due to their high surface area, abundant active sites, and large interlayer spacing for storing K ions.

An obvious choice is graphite as electrode, the successful material applied in Li ion batteries with formation of stage I LiC_6_ via intercalation. Graphite is electrochemically active to K ion intercalation, leading to the formation of fully potassiated stage I KC_8_ and a theoretical capacity of 278 mA h g^−1^.2d, 5 However, rate performance and cycling stability are still poor, and graphite suffers from large volume expansion (58 %) and a low K ion diffusion rate during the intercalation/extraction process.^5^, ^6^ Progress has been made to extend the stability to 500 cycles by expanding interlayer space and by nanostructure design in graphitic carbons.^7^ To pursue a long‐cycling lifespan with high rate capability, considerable efforts have been devoted to the exploration of novel carbonaceous materials. Progressive enhancement in cyclability reveals several critical structural parameters must be controlled for achieving a surface‐induced K‐adsorption mechanism beyond the intercalation chemistry, including developed pore nanostructures with high surface area and defects of heteroatom‐doped active sites.^8^ In this regard, N doping, particularly edge N (pyridinic/pyrrolic configurations) has been proved experimentally and theoretically to be efficient for adsorbing K ions with abundant electronegative defect sites and higher adsorption energy.8a, 9 Despite massive investigation, N species in carbons cannot be rationally tailored in edge‐enriched configurations, owing to trial‐and‐error strategies with uncontrolled N species involved in doping.8a, 8c, 10 Designing precursors with definite edge‐type N seems possible to promote edge‐enriched N in resulting carbons, such as using polypyrrole‐derived carbons with enhanced capacity and cyclability.^9^, ^11^ However, there is still enough room to further push forward the cycling performance with less decay rate (<0.002 % per cycle).9a, 12 The possible reasons for restricted cycling stability could be, on one hand, the incapability of rationally tailoring N species into edge‐enriched configurations. On the other hand, it is difficult to simultaneously achieve edge‐enriched N and high surface area, because of the well‐known trade‐off for both structural features.^13^ Moreover, little attention has been paid to the spatial N distribution or doping depth, which could also affect K storage performances. Thus, elaborate engineering of edge‐enriched N in carbonaceous materials is still highly desirable for achieving ultrastable cycling performance while maintaining high capacity and rate capability.

Herein, we demonstrate a new strategy to produce edge‐enriched N‐doped porous carbon nanosheets (ENPCS) for ultrastable K ion storage. The ENPCSs were fabricated by pyrolysis–etching of a pyridine‐coordinated polymer network, taking into account the predefined pyridinic N in this precursor. By lowering the pyrolysis temperature to 500 °C, we obtained ENPCS bearing both a high edge‐N content of 9.34 at % and a high surface area of 616 m^2^ g^−1^, as well a richer N distribution in the inner part of material. The ENPCS delivers a high reversible capacity (313 mA h g^−1^), high rate capability, and ultralong cycling stability with almost no degradation (0.0009 % decay rate and remaining capacity of 252 mA h g^−1^) over 6000 cycles at 1 A g^−1^, representing one of the best cycling stabilities. The high edge‐N content, richer N distribution inside the material, and high surface area are together responsible for its impressive ultrastable performance through full utilization of surface adsorption capacitive behavior. Our work constitutes an important step in deploying carbonaceous materials for stable K‐ion based energy storage systems.

## Results and Discussion

**Figure 1:**
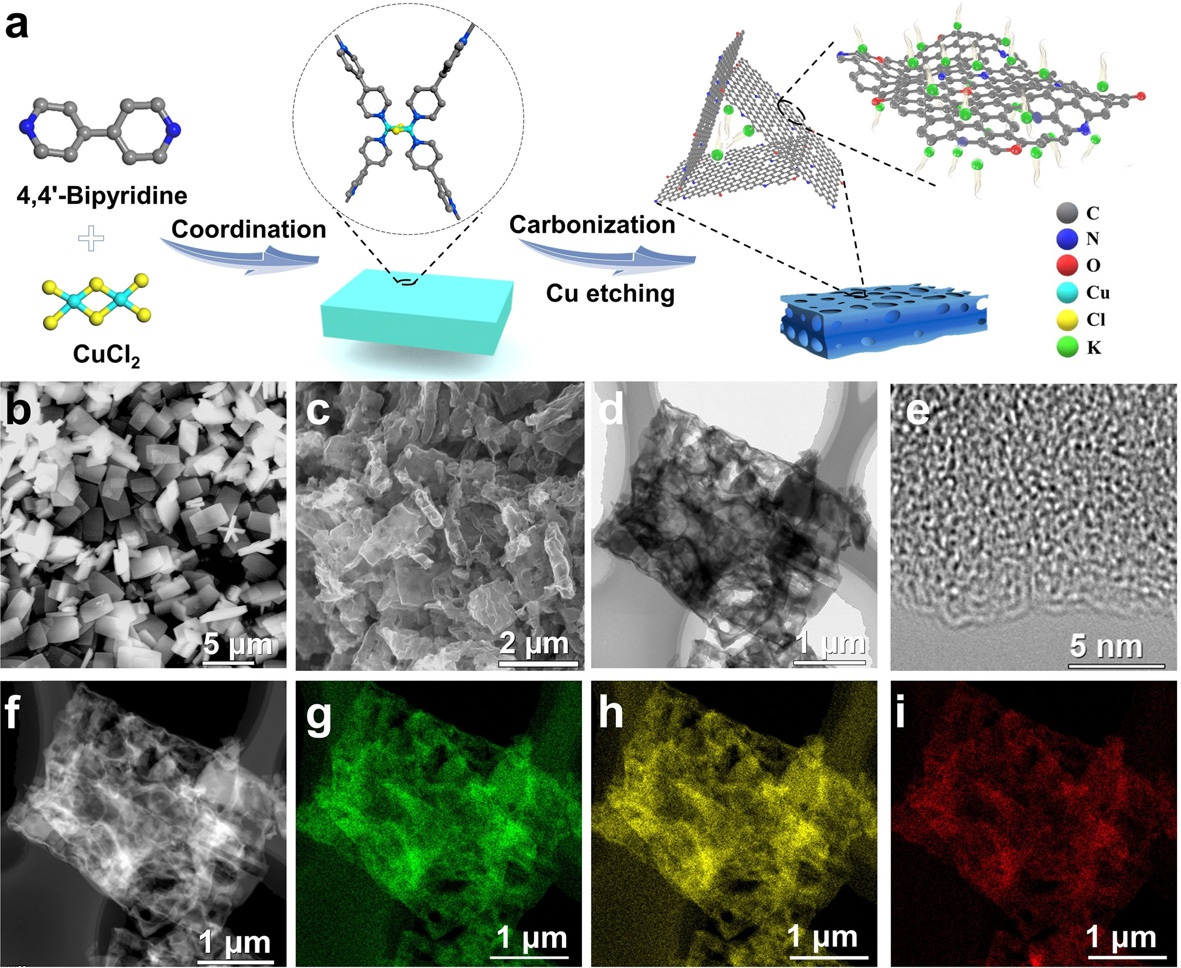
a) Schematic illustration of the synthesis of ENPCSs; SEM images of b) the precursor and c) resulting carbon sample ENPCS‐500 carbonized at 500 °C; TEM images of ENPCS‐500 with d) low and e) high magnification; f) HAADF‐STEM image and corresponding elemental mappings of g) carbon, h) nitrogen, and i) oxygen.

The fabrication of ENPCS (Figure 1 a) involves the fast coordination of bipyridine with copper ions for producing pyridine‐coordinated polymer network and subsequent pyrolysis and etching.^14^ The pyrolysis transforms aromatic pyridine moieties into edge‐N heterocyclic carbon rings fusing and stacking around copper species, while the etching enables the creation of microporosity by leaching the copper species, and meanwhile introduces some O functional groups. The polymer network itself shows a well‐defined cuboid‐like nanosheet morphology with a smooth surface (Figure 1 b). After carbonization at 500 °C, the resulting carbon ENPCS‐500 largely retains the precursor‐immanent morphology (Figure 1 c) with randomly aggregated cuboids. However, due to the shrinkage and pore creation effect, the surface becomes rough with many holes (Figure 1 c,d), indicative of the presence of structural defects that could be beneficial for K^+^ storage. The high‐resolution transmission electron microscopy (TEM) image of ENPCS‐500 reveals an amorphous structure with short turbostratic nanodomains, which are randomly packed and interconnected with rich microporosity (Figure 1 e). Some lattice fringes are observed with an interlayer distance of 0.38–0.41 nm (Figure 1 e), greater than that of graphite (0.335 nm). According to the HAADF‐STEM and element mappings, ENPCS‐500 exhibits an even distribution of N and O over the cuboids (Figure 1 f–i).

**Figure 2:**
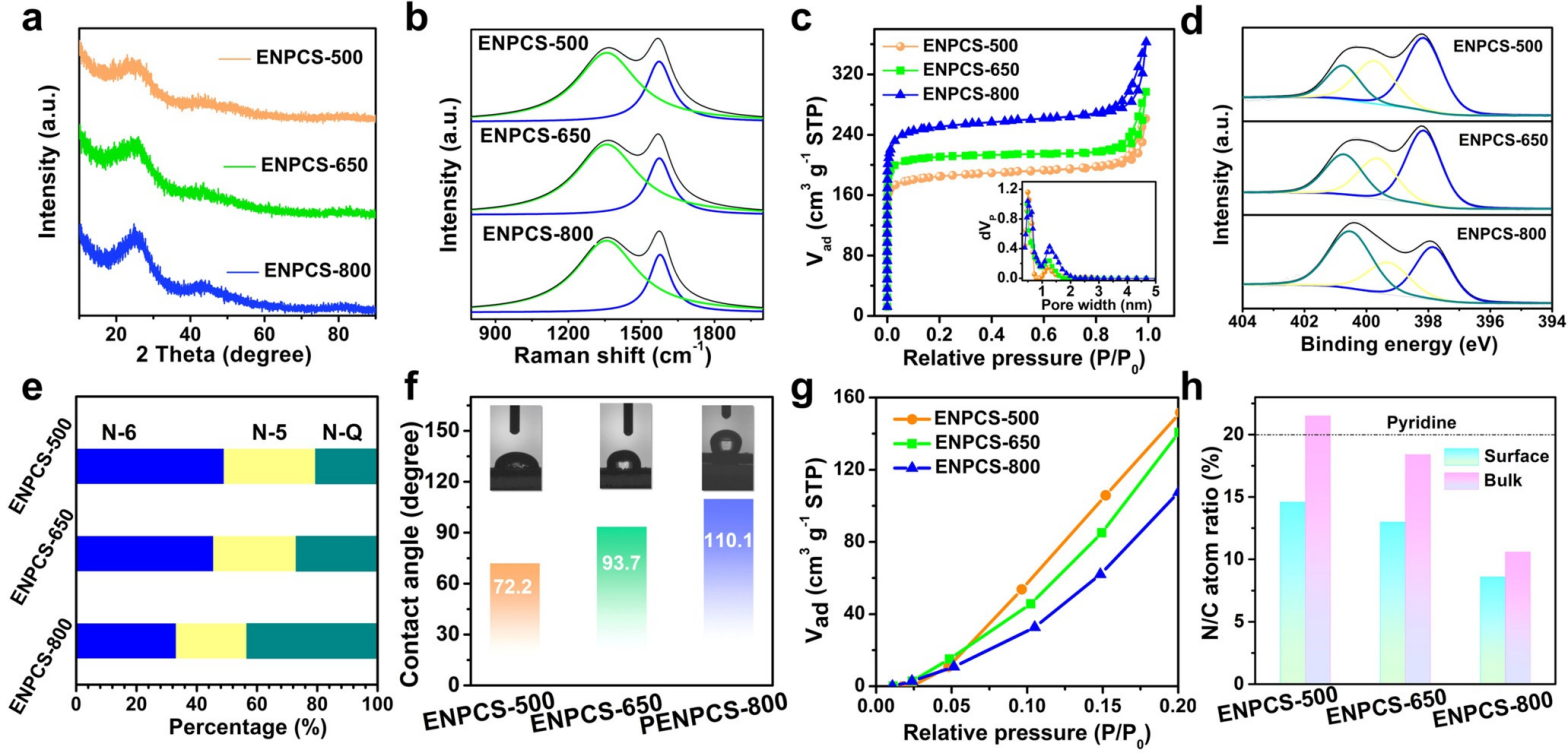
a) XRD profiles; b) Raman spectra; c) N_2_ adsorption/desorption isotherms (the inset shows the DFT pore size distribution curves); d) XPS high‐resolution N 1s spectra and e) corresponding percentage of N‐6, N‐5, and N‐Q; f) histograms of the contact angle of ENPCSs at 0.1 s, and the insets are the corresponding digital photos; g) water vapor adsorption isotherms at 298 K at low relative pressure; h) N/C atom ratio on the surface and in the bulk for ENPCSs.

With increasing pyrolysis temperature, ENPCS‐650 and ENPCS‐800 also reveal similar morphology features (Figure S1). However, the intrinsic crystallinity, heteroatom doping, and microporosity change. X‐ray diffraction (XRD) patterns with hump peaks of (002) and (100) diffractions suggest amorphous structures (Figure 2 a). The crystallinity degree was further assessed using an empirical *R* value.9b, 15 Higher pyrolysis temperatures give rise to increased *R* values from 1.91 for ENPCS‐500 to 2.03 and 2.32 for ENPCS‐650 and ENPCS‐800, respectively, manifesting the gradual development of a graphite‐like microcrystal structure. The intensity ratio of *I*_D_ (disorder‐induced D‐band) to *I*_G_ (in‐plane vibrational G‐band) in the Raman spectra for ENPCS‐500 has a value of 2.71, implying a disordered structure with many defects. Increased pyrolysis temperatures lead to higher *I*_D_/*I*_G_ values (Figure 2 b and Table S1). This is probably because at relatively lower temperature, ENPCS‐500 possesses a characteristic sp^2^‐hybridized hexagon structure inherited from the pyridinic precursor; higher temperatures promote the decomposition and conversion to amorphous carbons with defect nanopores/nanovoids.9b From N_2_ adsorption/desorption curves, ENPCSs exhibit predominately micropores with a narrow pore size around 0.5 nm and a less intense peak at 1.2 nm (Figure 2 c). A Brunauer—Emmett–Teller (BET) surface area up to 616 m^2^ g^−1^ was obtained for ENPCS‐500. Higher temperature results in enhanced microporosity and surface area, which is probably ascribed to the gradual loss of carbon and doped heteroatoms at elevated temperatures, forming more defects in the carbon structure.

ENPCSs are composed of C, N, and O according to the X‐ray photoelectron spectroscopy (XPS) spectra (Figure S2). The N atomic content is 11.8 %, 10.6 %, and 7.4 % for ENPCS‐500, ENPCS‐650, and ENPCS‐800, respectively, and the corresponding N:C ratio decreases, implying a more significant decomposition rate for N than for C at high temperatures (Table S2). Further N 1s spectra show that pyridinic N (N‐6, ≈398.1 eV) in the precursor splits into pyrrolic N (N‐5, ≈399.7 eV) and graphitic N (N‐Q, ≈400.7 eV) (Figure 2 d and Table S3). ENPCS‐500 delivers the highest edge‐N (N‐6/N‐5) ratio of 79.4 %, while this value decreases to 73.0 % and 56.6 % for ENPCS‐650 and ENPCS‐800, respectively (Figure 2 e). As a result of both the high N doping level and the high edge‐N ratio, ENPCS‐500 demonstrates an actual edge‐N content of 9.34 at %, superior to ENPCS‐650 (7.75 at %), ENPCS‐800 (4.20 at %), and the majority of reported N‐doped carbons.7c, 8b, 8c, 9, 12, 16 Although a few carbons favor higher edge N, they are prone to result in low surface area8f, 17 or vice versa.16a For example, when edge N exceeds 6 at %, the surface area is generally below 500 m^2^ g^−1^ (Table S4). The majority of the N‐doped carbons require a higher carbonization temperature (≥600 °C), because further lower temperature leads to incomplete carbonization with reduced conductivity or heteroatom doping.8f, 10b, 18 This is likely due to the intrinsic properties of the precursors.9b The edge‐enriched N and high surface area will be beneficial for the interfacial adsorption for capacitive process.

The surface content of O also decreases with increasing carbonization temperature (Table S2), which can be deconvoluted into three peaks for C=O, C−OH or C−O−C, and COOH (Table S5). C 1s spectra with C=C/C−C, C−O/C−N, C=N, and C=O were recorded (Table S6). The high N‐ and O‐doping is also responsible for the enlarged lattice spacing (Figure 1 e), and could make the surface more polar and hydrophilic.^19^ The dynamic contact angle (DCA) measurements can reflect the surface characteristics to a large extent for porous materials, once the water drops start to contact the surface. ENPCS‐500 always shows the lowest DCA at intervals between 0.1 and 0.4 s (Figure 2 f, Figures S3 and S4), demonstrating its high N‐ and O‐decorated surface. Likewise, the superior low‐pressure water capture of ENPCS‐500 also indicates the higher N‐ and O‐content and enhanced hydrophilicity (Figure 2 g). In contrast, the total capacity of ENPCS‐500 is lower (Figure S5), ascribed to the larger surface area and pore volume of ENPCS‐650 and ENPCS‐800.

The bulk elemental composition was measured by combustion elemental analysis. The bulk N:C atom ratio decreases with increasing pyrolysis temperature, consistent with the conclusion from XPS (Table S2). Intriguingly, it is found that ENPCS‐500 bears a bulk N:C atom ratio of 1:4.7, higher than that (1:6.8) determined by XPS and even the value of pyridine rings in the precursor (1:5) (Figure 2 h). Considering XPS is a surface technique with a penetration depth of several nanometers, the higher bulk N:C ratio indicates that the distribution of N is more concentrated at the interior of the cuboids. This probably originates from the thermally induced contraction and compression at the nanointerface between the organic pyridine moieties and the copper species template, thus retarding the decomposition of N inside.^20^ The removal of the template could expose more edge N atoms at defect sites like nanopore walls at inner materials. For the other two carbons obtained at higher temperatures, similarly higher bulk N:C ratios were also observed, but with significant decreased values. Such a gradient‐like distribution of N could act as a reservoir for exposing renewed active N sites upon continuous cycling. Taken together, these results clearly suggest that the use of an aromatic pyridine‐coordinated polymer network and a lower pyrolysis temperature simultaneously achieved edge‐enriched N content of 9.34 at %, richer N distribution inside the defect sites of the materials, and a large surface area (616 m^2^ g^−1^), which are expected to facilitate the surface‐dominated capacitive storage.^21^

**Figure 3:**
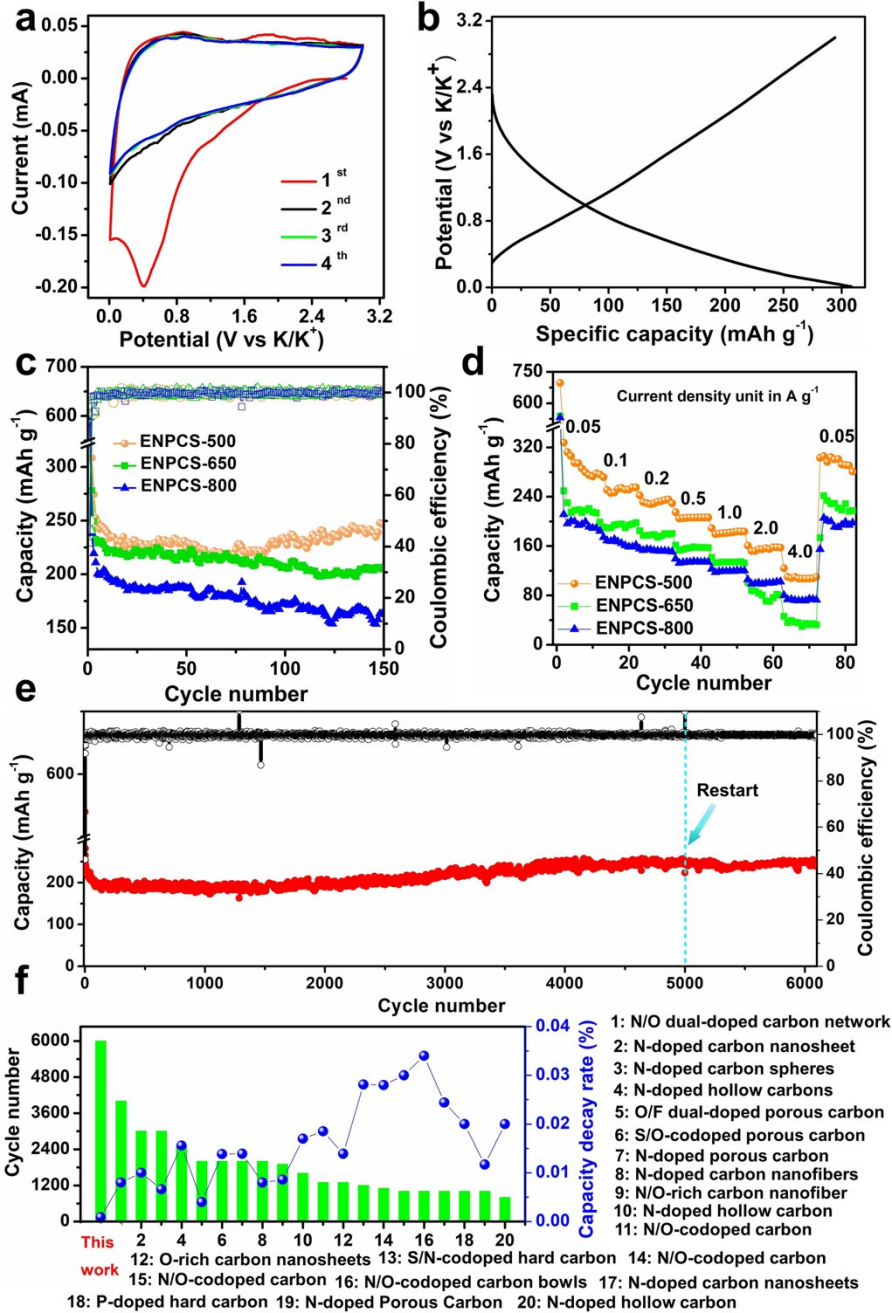
a) CV curves of ENPCS‐500 at 0.1 mV s^−1^; b) the second galvanostatic discharge–charge curve of ENPCS‐500 at 0.05 A g^−1^; c) cycle performances and Coulombic efficiency at 0.1 A g^−1^ and d) rate performances with 10 cycles at each rate; e) long‐term cycling performance and Coulombic efficiency of ENPCS‐500 at high rate of 1 A g^−1^. The cell was restarted from 5000 cycle; f) comparisons of cycle number and corresponding average capacity decay rate per cycle of various heteroatom‐doped carbons. Note: for (c)–(e), the initial two cycles were performed at 0.05 A g^−1^.

Figure 3 a presents the initial four cyclic voltammetry (CV) curves of ENPCS‐500. A cathodic peak at 0.41 V in the initial cycle disappears in subsequent cycles, probably because of the formation of a solid electrolyte interface (SEI) film. Another small peak near the cutoff voltage (0.01 V) is associated with the K^+^ intercalation. Upon charging, a hump peak around 0.87 V corresponds to the depotassiation process. The curves overlap well after the second scan, suggesting a highly reversible electrochemical behavior. In spite of similar CV characteristics for ENPCS‐650 and ENPCS‐800, a progressive reduction in areas of the CV curves was observed (Figure S6), revealing a decreased capacity. This is mainly caused by the elimination of N species at higher temperature. Galvanostatic discharge–charge curves show behaviors in keeping with the CV results, and an initial discharge capacity of 628 mA h g^−1^ and a reversible charge capacity of 313 mA h g^−1^ were obtained for ENPCS‐500, corresponding to an initial Coulombic efficiency (ICE) of 50 % (Figure S7a). The low ICE is frequently observed for amorphous carbonaceous materials. The ICE of ENPCS‐500 is higher than that of most reported N‐doped carbons.8a, 8d, 8h, 16a, 17, 18, 22 However, further work should be focused on reducing the irreversible capacity such as design of novel structures, prepotassiation, and optimization of electrolyte.16a ENPCS‐650 and ENPCS‐800 show a decreased ICE of 46 % and 41 %, respectively (Figure S7b,c), probably due to the increased surface area. A typical sloping discharge–charge curve was observed after the first cycle (Figure 3 b), revealing a capacitive‐dominated K^+^ storage behavior.8d, 9b, 21 The capacity mainly derives from 0.1–1.6 V, which could be beneficial for alleviating the risk of dendrite formation for safety.^21^, ^23^ The Coulombic efficiency reaches 95 % at the second cycle and then gradually stabilizes to 99 % after about ten cycles for ENPCSs (Figure 3 c), demonstrating a good reversibility.

After two cycles at 0.05 A g^−1^, cells were subjected to further cycling at 0.1 A g^−1^. ENPCS‐500 shows a slight capacity loss in the initial 15 cycles and then a gradual increase to 246 mA h g^−1^ after 150 cycles (Figure 3 c). However, ENPCS‐650 and ENPCS‐800 show continuous fading with residual capacities of 206 and 166 mA h g^−1^ after 150 cycles, respectively. The capacity decay in the initial cycles could be attributed to the incomplete SEI formation as well as the irreversible decomposition of electrolyte on the high‐surface‐area carbon.^24^ The gradual increase of ENPCS‐500 afterwards is probably caused by the progressive utilization of N‐rich domains located inside the particles and deeper penetration during cycling. In contrast, ENPCS‐650 and ENPCS‐800 show significantly reduced N doping, giving rise to little increase or even decrease in capacity. ENPCS‐500 shows the best rate capabilities (Figure 3 d), delivering reversible capacities of 276, 255, 229, 206, 184, 157, and 110 mA h g^−1^ at current densities of 0.05, 0.1, 0.2, 0.5, 1.0, 2.0, and 4.0 A g^−1^, respectively. When the rate turns back to 0.05 A g^−1^, the capacity is entirely recovered, indicative of high reversibility and excellent structural integrity. In contrast, ENPCS‐650 and ENPCS‐800 retain lower capacities of 32 and 73 mA h g^−1^, respectively at a high rate of 4.0 A g^−1^. The rate performance of ENPCS‐500 is comparable to or even better than that of some reported carbons (Figure S8). Generally, the rate performance is governed by both the electronic and ionic conductivity. The former is mainly influenced by the carbonization temperature, whereby a higher temperature will increase the conductivity.^18^ The latter, associated with ion transport/diffusion, is governed by doped active sites and porous structure. Considering that both the lower carbonization temperature and surface area are thought to be negative to electronic and ionic conductivity, respectively, ENPCS‐500 still delivers superior rate performance, again highlighting the critical role of edge‐enriched N for facilitating the ion transport/diffusion.

The long‐term cycling of ENPCS‐500 was tested at 1 A g^−1^ (Figure 3 e). After the first two cycles at 0.05 A g^−1^, ENPCS‐500 was subjected to cycling at 1 A g^−1^ and shows a capacity fading from 266 to 183 mA h g^−1^ at 137 cycles at 1 A g^−1^. Then the capacity stabilized and a capacity of 192 mA h g^−1^ was obtained after about 2000 cycles (Figure S9). Subsequently, a gradual rise was observed and the capacity reached 247 mA h g^−1^ after 5000 cycles, very close to its initial capacity at 1 A g^−1^. The stabilization during the 2000 cycles indicates the wetting of the surface or near surface, enhancing the reversibility of the charge storage. The continuous rise afterwards is mainly related to further utilization of the richer N distribution inside the material, which serves as an N reservoir to release more active N sites for capturing K ions. The result suggests the importance of constructing gradient‐like N distribution for ultrastable cycling. This phenomenon can also be reflected by electrochemical impedance spectra (Figures S10 and S11), in which the calculated charge transfer resistance (*R*_ct_) gradually decreases and then stabilizes (*R*_ct_ is 1902, 1522, 1581 Ohm at 100, 1500, 2000 cycles, respectively, Table S7). When the cell was restarted after 5000 cycles, there was still no decay (252 mA h g^−1^ to 6000 cycles), further revealing the robust structure. A capacity retention of 94.5 % can be obtained during the whole 6000 cycles at 1 A g^−1^, corresponding to a capacity decay rate of only 0.0009 % per cycle (Figure 3 f). Such a low attenuation rate is rarely observed for carbonaceous materials including heteroatom‐doped carbons (Figure 3 f), other porous carbons, and even some carbon composites with metallic compounds (Table S8), validating the advantage of such an architecture design on alleviating structural degradation. The cuboid‐like structure is well retained after 1000 cycles (Figures S12 and S13a). Elemental mapping still displays a homogeneous distribution of C, N, O, and K over the cuboids (Figure S13b–f), confirming the remarkable structural and N‐doping stability for effective K storage. In contrast to intercalation chemistry, the surface‐driven pseudocapacitive behavior mainly occurs at edges, defect sites, or pore surface, without causing damage to the electrode materials. ENPCS with high edge‐enriched N doping, defect structure, and high surface area could enable the full utilization of pseudocapacitive behavior, as discussed below with ENPCS‐500 and ENPCS‐800.

**Figure 4:**
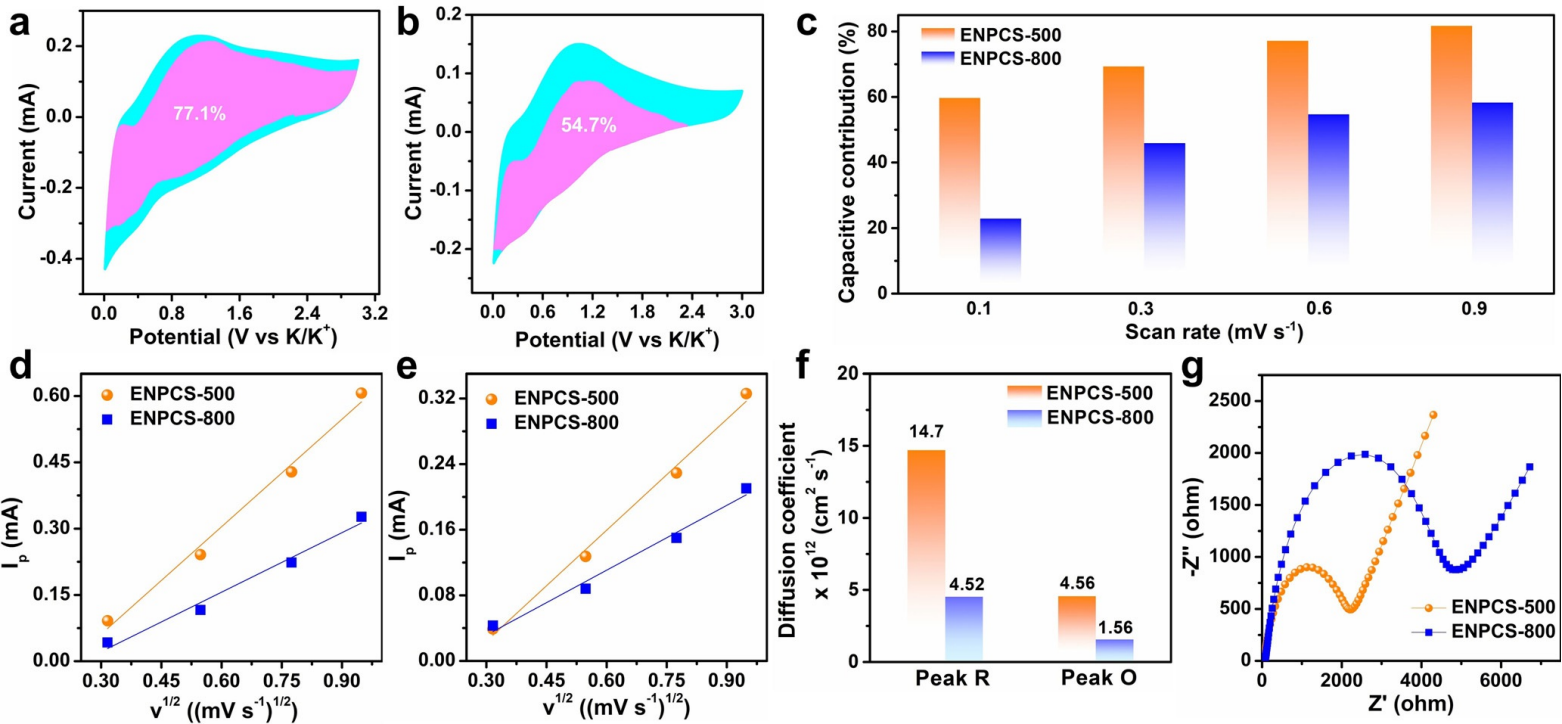
The separation of capacitive (pink region) and diffusion‐controlled (cyan region) contribution at 0.6 mV s^−1^ for a) ENPCS‐500 and b) ENPCS‐800; c) capacitive contribution at different scan rates; the plots of current versus square root of the scan rate of d) reduction peak, e) oxidation peak, and f) the corresponding K ion diffusion coefficients; g) EIS of ENPCS‐500 and ENPCS‐800 after 100 cycles at 1 A g^−1^.

The kinetics were investigated by conducting CV tests with different scan rates (Figures S14a and S15a). According to the equation *i*(V)=*k*_1_ *v* + *k*_2_ *v* ^1/2^, *i* is separated into a surface‐driven pseudocapacitive process (*k_1_v*) and a diffusion‐controlled contribution (*k*_2_ *v* ^1/2^). Figure 4 a,b show the contributions from pseudocapacitive and diffusion processes at 0.6 mV s^−1^. A predominately capacitive contribution of 77.1 % is achieved for ENPCS‐500, significantly higher than 54.7 % for ENPCS‐800. By analysis of other scan rates (Figures S14b–d and S15b–d), the capacitive contribution improves with higher scan rates. ENPCS‐500 always shows higher capacitive contribution than ENPCS‐800 (Figure 4 c). Even at a very low scan rate of 0.1 mV s^−1^, a higher capacitive‐dominated contribution of 59.7 % is obtained for ENPCS‐500, whereas ENPCS‐800 is characterized by a diffusion process with a low capacitive contribution of 22.9 %. Such pseudocapacitive behavior can be enhanced by increasing the surface area or introducing defects such as N‐doping sites. The simultaneous achievement of edge‐enriched N and high surface area in ENPCS‐500 (Table S4) is beneficial for the highly efficient pseudocapacitive process. Surface O‐containing groups could contribute to the performance,8g, 9a, 25 generally around 1.7 V for adsorbing K ions. The much lower capacity contribution around 1.7 V and the relatively low O content result in much less capacity from O. The higher level of edge‐N sites and developed porosity induce more defects, leading to fast K ion diffusivity kinetics. The diffusion coefficient, *D*_k_ was calculated by the Randles–Sevcik equation, showing linear plots with higher slope for ENPCS‐500 (Figure 4 d,e). *D*_k_ is 1.47×10^−11^ and 4.56×10^−12^ cm^2^ s^−1^ for reduction and oxidation peaks, respectively, greater than those for ENPCS‐800 (Figure 4 f). Likewise, ENPCS‐500 demonstrates smaller *R*_ct_ (Figure 4 g and Table S7). Consequently, the superior K ion diffusivity kinetics in ENPCS‐500 is responsible for its high capacity and rate performance.

**Figure 5:**
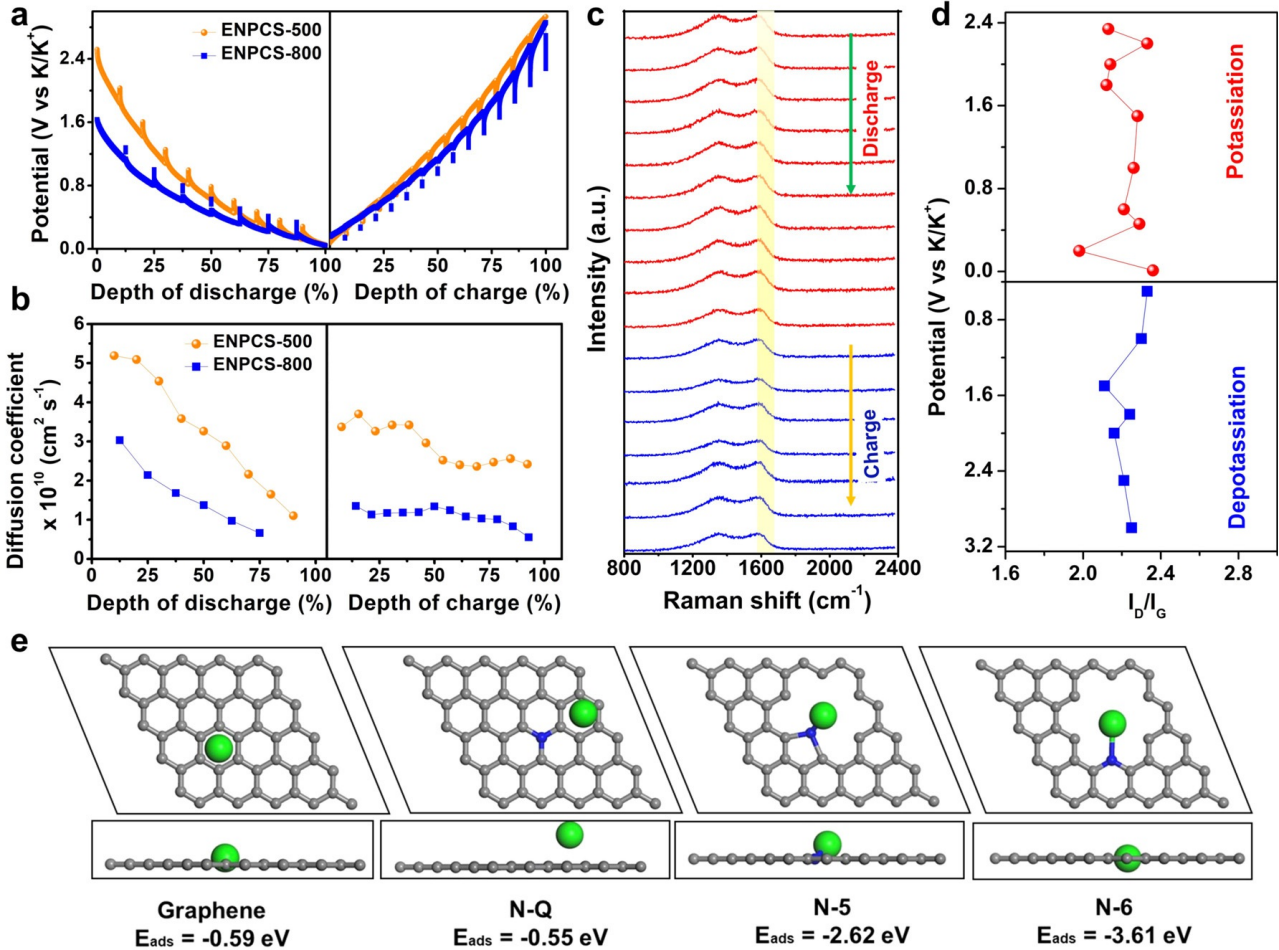
a) GITT profiles and b) the corresponding calculated diffusivity curves for ENPCS‐500 and ENPCS‐800; c) in situ Raman spectra and d) the corresponding potential vs. *I*_D_/*I*_G_; e) theoretical simulation for K adsorption energy with graphene and different N‐doping defect sites.

Galvanostatic intermittent titration (GITT) measurements were further performed to elucidate the diffusion kinetics of K ions during the dynamic potassiation/depotassiation process (Figure 5 a). ENPCS‐500 exhibited smaller overpotentials compared to ENPCS‐800, indicating a higher *D*_k_. The resulting *D*_k_ as a function of the depth of discharge–charge was calculated, and ENPCS‐500 shows higher *D*_k_ than ENPCS‐800 during the whole potassiation and depotassiation process (Figure 5 b), manifesting its faster K ion diffusivity. Considering that the surface area of ENPCS‐500 is slightly lower than that of ENPCS‐800 (616 vs. 842 m^2^ g^−1^), one may suggest that favorable K ion diffusivity is mainly dominated by the accessible edge‐enriched N sites. A progressive decline of *D*_k_ was observed as potassiation proceeds, mainly because K ions have to overcome the repulsive gradient from previously adsorbed K ions.22c In situ Raman spectra were recorded to validate the pseudocapacitive dominated charge storage property (Figure 5 c). Previous studies indicate that the G‐band or the intensity ratio of *I*_D_/*I*_G_ could be employed to monitor the staging behavior.8a, 8f, 26 The intercalation of K ions into graphitic layer generally results in a change of *I*_D_/*I*_G_, weakening and even disappearance of the G and D bands at the fully discharged state due to the formation of KC_*x*_.10a, 27 However, it can be seen that the D and G bands remain almost unaffected (Figure 5 c,d), demonstrating that the storage of K ions does not induce structural changes, which further supports the surface‐adsorption mechanism. A similar observation was reported for non‐graphitic carbon even at higher temperature.10a The result can also explain the near‐sloped line in the discharge–charge curve (Figure 3 b) with predominate surface‐driven process and minor potassium intercalation,8d, 24 in contrast to graphite which displays a plateau region below ≈0.2 V. Although such a slope‐dominated process could avoid the possible plating of K metal for safety purpose, the relatively high potential without a plateau will decrease the energy density of the full cell. Studies are in progress to construct some local expanded graphitic structures in such defect edge‐enriched N carbon layers for practical applications.

To theoretically reveal the superior K ion storage capability by edge‐enriched N, the relative K adsorption abilities on carbon lattice with various N configurations were simulated (Figure 5 e). The adsorption energy (*E*_ads_) of K atoms on an idealized graphene layer is calculated to be −0.59 eV, and *E*_ads_ on an N‐Q site is −0.55 eV. For the edge N doping, we can envisage, ideally but reasonably, that two situations can be considered: one is the location at the material outer surface (edge plane of carbon rings) and the other is interior of the materials such as defects of carbon basal planes (e.g., pore walls and nanovoids). For the former, the *E*_ads_ for N‐6 is −1.16 and −1.01 eV with two possible sites, and the *E*_ads_ for N‐5 is −1.55 eV (Figure S16). The result indicates that N‐5 shows higher adsorption ability than N‐6 in this case, but both are superior to the N‐Q site. The higher *E*_ads_ of the N‐Q site originates from the electron‐rich structure with a negative effect on K adsorption. For the latter, the *E*_ads_ for N‐5 and N‐6 at defect sites inside materials is determined to be −2.62 and −3.61 eV, respectively, significantly lower than that of N‐Q and those N atoms at the edge plane of carbon rings on the material outer surface. ENPCSs exhibit similar pores with small sizes at 0.5 nm, enabling concentrated edge‐located N on the defect pore surface. The much higher content of edge‐enriched N of ENPCS‐500 thus exhibits superior capacity and high rate capability than other ENPCSs. More interestingly, much richer N distribution (N:C>1:4.7) within ENPCS‐500 also makes continuous utilization of N‐6/N‐5 at defect sites inside possible with a stronger tendency for K capturing, resulting in a gradual increase of capacity with an outstanding cycling performance. These results vividly show that it is vital to increase the edge‐N doping level, especially at the defect carbon layer sites to maximize the K adsorption and cycle stability.

## Conclusion

In summary, we demonstrated the preparation and utilization of edge‐enriched N‐doped porous carbons for fast and stable K ion storage. The pyrolysis–etching of a pyridine‐coordinated polymer enables the conversion of pyridine moieties into edge‐enriched N active sites at lower carbonization temperature, and the generation of micropores with rich defects via etching. The optimized sample with superior edge N obtained at 500 °C displays high capacity, excellent rate capability, and robust cycle stability, thanks to the surface‐dominated pseudocapacitive storage characteristic with fast ion diffusivity. Most noteworthy is the extraordinary cyclability with 0.0009 % capacity decay rate for 6000 cycles at 1 A g^−1^. This is also related to the unique structure with richer N distribution inside the defect sites of the materials, acting as a reservoir to expose successively renewed N sites upon cycling. Theoretical simulations show the much higher adsorption energy for edge N at the defect sites inside the materials. The utilization of defect edge‐N active sites with controlled spatial distribution could be valuable for various carbon‐based electrode systems in the future.

## Conflict of interest

The authors declare no conflict of interest.

## Supporting information

As a service to our authors and readers, this journal provides supporting information supplied by the authors. Such materials are peer reviewed and may be re‐organized for online delivery, but are not copy‐edited or typeset. Technical support issues arising from supporting information (other than missing files) should be addressed to the authors.

Supplementary
