# Supplementary material for: Ultrastable Surface‐Dominated Pseudocapacitive Potassium Storage Enabled by Edge‐Enriched N‐Doped Porous Carbon Nanosheets
Source: Angew Chem Int Ed Engl. 2020 Jun 8;59(44):19460–7. doi: 10.1002/anie.202005118 (PMC7687278; doi:10.1002/anie.202005118)
Supplement: Supplementary file 1 — Supplementary [file ANIE-59-19460-s001.pdf]

Angewandte Chemie
Eine Zeitschrift der Gesellschaft Deutscher Chemiker

Supporting Information

Ultrastable Surface-Dominated Pseudocapacitive Potassium Storage Enabled by Edge-Enriched N-Doped Porous Carbon Nanosheets

Fei Xu, Yixuan Zhai, En Zhang, Qianhui Liu, Guangshen Jiang, Xiaosa Xu, Yuqian Qiu, Xiaoming Liu, Hongqiang Wang, and Stefan Kaskel**

anie_202005118_sm_miscellaneous_information.pdf

Table of Contents

1. Experimental Procedures
2. Supporting Figures
3. Supporting Tables
4. Supporting References

1. Experimental Procedures

Materials synthesis.
Preparation of pyridine-coordinated polymer network. Typically, 5.6 mmol L^{-1} $CuCl_2{\cdot}2H_2O$ aqueous solution was prepared as solution A. 1.0 g triblock copolymer F127 was dissolved in 100 mL 0.1 mol L^{-1} 4, 4'-bipyridine water-ethanol (volume ratio of 1:17) solution under constant stirring, denoted as solution B. Then, 100 mL solution B was added into 900 mL solution A. After a rapid mixing (30 s), the resultant products were collected by centrifugation under ca. 5000 rmp for 7 min and washed with deionized water for three times. The collected light blue polymers were then dried under 80 ºC.
Preparation of ENPCSs. The obtained carbon precursor was carbonized in a tube furnace at 500, 650 and 800 ºC (denoted ENPCS-500, ENPCS-650 and ENPCS-800) for 2h with a heating rate of 1 ºC min^{-1} under N_2 atmosphere. Black carbon products were collected after carbonization and then immersed in 10 mL 4 mol L^{-1} HNO_3 for 24 h to remove copper species. Finally, the black carbon products were washed with deionized water to remove acid and then dried under vacuum at 80 ºC for 24 h. Due to the hydrophilicity feature, all ENPCSs were stored in drying vessels.
Material characterization. The morphology of the ENPCSs was characterized with field-emission scanning electron microscope (FEI NANOSEM 450) and transmission electron microscope (FEI Talos F200X). The porosity feature was examined using N_2 adsorption-desorption isotherms on a Micromeritics ASAP 2020 and the Brunauer-Emmett-Teller (BET) surface area was based on BET theory. The pore size distribution was analyzed by original density functional theory (DFT) combined with non-negative regularization and medium smoothing. The dynamic contact angle test was performed on a SL200KB optical contact angle & interface tension meter (Kino, USA). Wide-angle powder X-ray diffraction patterns (XRD) were conducted on a Shimadzu XRD-7000 (Cu Kα radiation, 0.15406 nm). Raman spectroscope measurements were performed in a Renishaw inVia Raman spectroscope with a 532 nm laser. XPS spectra were acquired with a Shimadzu Kratos Supra with an Al Kα monochromatic source. Water physisorption measurements were carried out on a Quantachrome Hydrosorb 1000 at 25 ºC. Prior to measurements the samples were degassed at 150 °C for at least 12 h.
Cell fabrication and K ion storage performance. The electrodes were fabricated by mixing ENPCS, Super P and binder of sodium carboxymethyl cellulose (1.5 wt%) and styrene butadiene rubber (40 mg mL^{-1}) aqueous solution at a weight ratio of 70:20:8:2 and then the slurry was coated uniformly on a copper foil. The mass loading of active material ENPCS is controlled around 0.8 mg cm^{-2} in each electrode. The electrodes were dried at 70 ºC under vacuum for at least 8 h and then cut into a round disk with diameter of 10 mm. Further drying with each disk was performed at 80 ºC until the weight remains constant. The half-cell electrochemical measurements were carried out in 2032-type coin cells assembled in an Ar-filled glovebox with K foil as the counter electrode and 1 M KFSI in ethylmethyl carbonate as the electrolyte solution. The cyclic voltammogram (CV) with a scan rate of 0.1-0.9 mV s^{-1} and electrochemical impedance spectroscope (EIS) were performed on a CHI606E electrochemical workstation (Shanghai Chenhua). Galvanostatic discharge-charge (0.01-3V) and galvanostatic intermittent titration technique (GITT) measurements were conducted on a Land CT 2001A battery testing system (Land, China). The GITT measurements consisted of application of a current pulse of 0.05 A g^{-1} for 30 min, followed by relaxation for 3 h. The in situ Raman spectra was collected on Renishaw inVia Raman spectroscope combined with CHI606E electrochemical workstation at scan rate of 1 mV s^{-1} a specialized self-made coin cell.
Calculation of ion diffusion coefficient from CV curves at different scan rates.
According to the Randles-Sevcik Equation as show by

$$I_p = 2.69 \times 10^5 \times n^{1.5} A \times D_K^{0.5} \times v^{0.5} \times C$$

Where I_p represents the peak current (A), n is the number of electrons transferred, A is the electrode area (cm^{-2}), v is the corresponding scan rate, and C is the bulk concentration (mol cm^{-3}).[1] From the linear relationship of I_p and $v^{0.5}$, the diffusion coefficients D_{K1} (k_1 corresponds to reduction peak), D_{k2} (k_2 corresponds to oxidation peak) were calculated, as shown in Fig. 4c.
Electrochemical impedance spectroscope simulation.
The impedance data is simulated by electric equivalent circuit as followed:

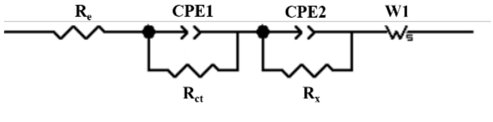

where R_e represents the electrolyte resistance; R_{ct} and CPE1 correspond to charge-transfer resistance and double-layer capacitance; R_x and CPE2 are the resistance and capacitance of the surface film fabricated on the electrodes, respectively; W_1 stands for the Warburg impedance.[2, 3] The corresponding parameters are listed in Table S7.

Galvanostatic intermittent titration (GITT) measurement.

The diffusion coefficients of K-ions in ENPCS electrodes were measured with a periodic repetition of a current pulse of 50 mA g^{-1} for 30 min, followed by relaxation for 3 h. The dependence of diffusion coefficient on the depth of discharge/charge were calculated with the following equation:[4]

$$D = \frac{4}{\pi\tau}\left(m_B \frac{V_m}{M_b\, S}\right)^2 (\Delta Es/\Delta Et)^2$$

τ is the duration of the current impulse; m_B is the mass load of the electrode materials; S represents the geometric area of the electrode. ΔEs is the quasi-thermodynamic equilibrium potential difference between before and after the current pulse; ΔEt represents the potential difference during the current pulse; V_m is the molar volume of the materials; and M_b is the molar mass of carbon. The relative value of M_b/V_m can be calculated from the density of materials using the following equation:

$$\rho = \frac{1}{V_{total} + 1/\rho_{carbon}}$$

ρ (g cm^{-3}) is the density of materials, V_{total} (cm^3 g^{-1}) is the total pore volume analyzed from the N_2 isotherm, and ρ_{carbon} is the true density of carbon (2 g cm^{-3}). The calculated diffusion coefficients are plotted as function of discharge/charge depth in Fig. 4f.

Theoretical calculations of different N configurations on K ions.

We have employed the Vienna Ab Initio Package (VASP) [5] [6] to perform all the density functional theory (DFT) calculations within the generalized gradient approximation (GGA) using the PBE[7] formulation. The projected augmented wave (PAW) potentials [8] [9] were chosen to describe the ionic cores and valence electrons were taken into account using a plane wave basis set with a kinetic energy cutoff of 400 eV. Partial occupancies of the Kohn−Sham orbitals were allowed using the Gaussian smearing method and a width of 0.05 eV. The electronic energy was considered self-consistent when the energy change was smaller than 10^{-7} eV. A geometry optimization was considered convergent when the energy change was smaller than 10^{-6} eV. The spin polarization was considered in all calculations.

The equilibrium lattice constant of hexagonal graphene unit cell was calculated, when using a gamma-point centered 15×15×11 k-point grid for Brillouin zone sampling, to be a=b=2.4680 Å. This unit cell was used to construct the monolayer graphene with a *p*(9×6) periodicity in the x, y directions, separated by a 15 Å of vacuum in the z direction between the slab and its periodic images. Then the left 4 columns of C atoms were deleted to create the edge atoms, which makes the graphene with 60 C atoms remained. The two-coordinated edge C atoms were saturated by H atoms in order to passivate the edge C atoms which would be falsely active. The gamma k-point in the Brillouin zone was used in the following surface calculations. During structural optimizations, all atoms were allowed to fully relax.

The adsorption energy of a K atom was defined as: $E_{ads} = E_{K/surf} - E_{surf} - E_K$, where $E_{K/surf}$, E_{surf} and E_K are the energy of the K adsorbed on the graphene surface, the energy of graphene clean surface, and half of the energy of body-centered cubic K unit cell (containing 2 K atoms), respectively.

2. Supporting Figures

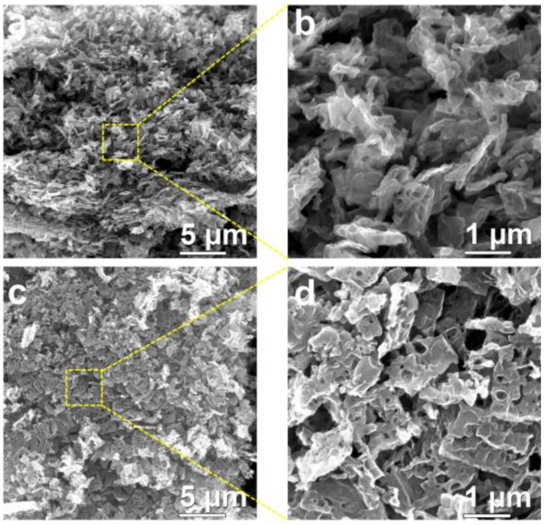

Figure S1. SEM images of (a, b) ENPCS-650, and (c, d) ENPCS-800 with low and high magnifications.

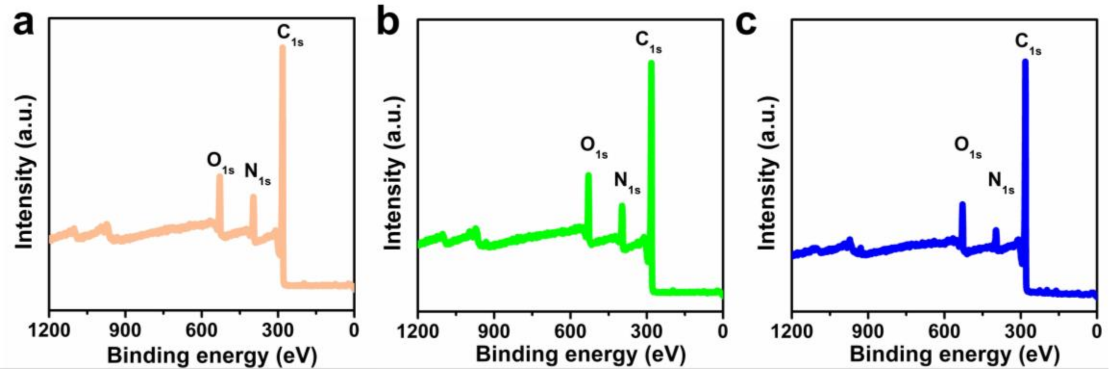

Figure S2. XPS survey spectra of (a) ENPCS-500, (b) ENPCS-650 and (c) ENPCS-800.

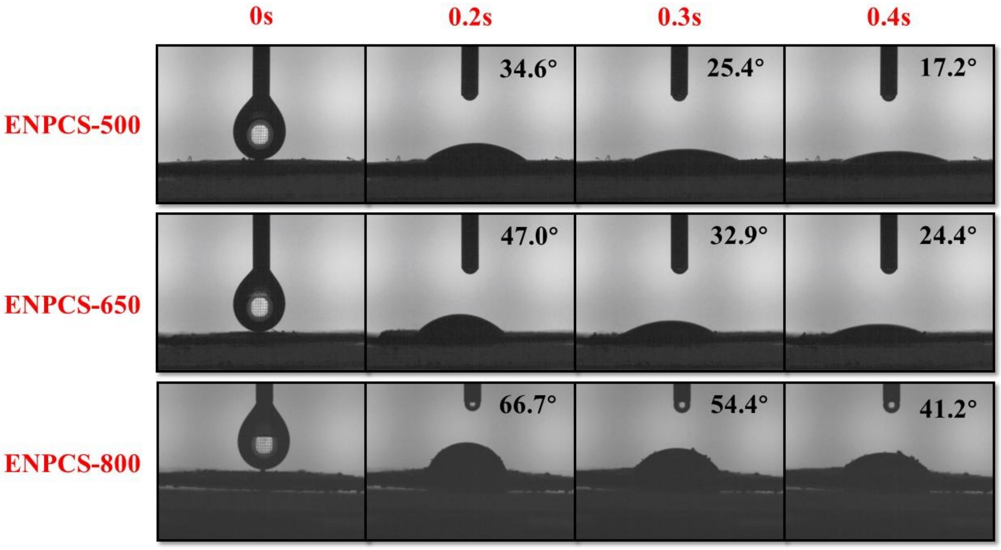

Figure S3. Digital photos of dynamic contact angle from 0 to 0.4s for all ENPCSs.

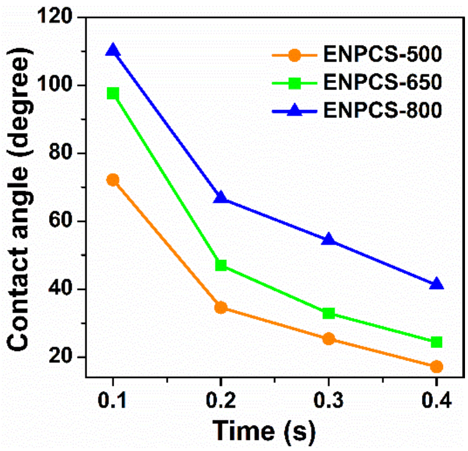

Figure S4. Dynamic contact angles as a function of measured time from 0.1 to 0.4 s.

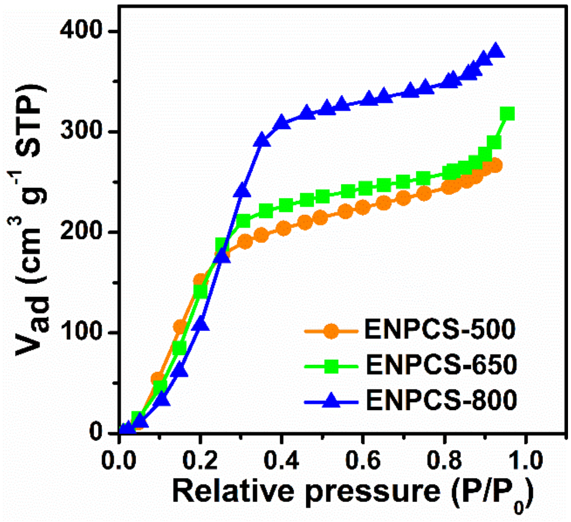

Figure S5. Water vapor adsorption isotherms at 25 ºC.

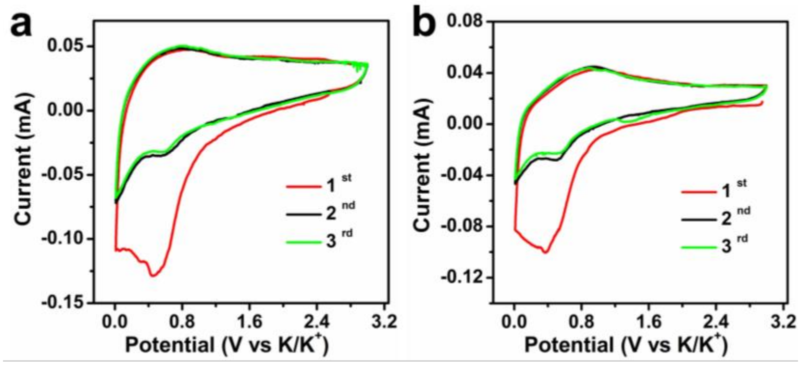

Figure S6. CV curves of (a) ENPCS-650 and (b) ENPCS-800 for the first three cycles at 0.1 mV s^{-1}.

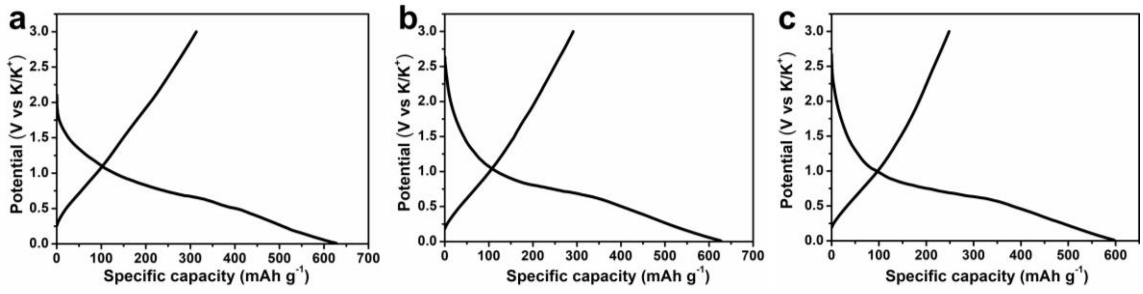

Figure S7. Initial galvanostatic discharge-charge curves of (a) ENPCS-500, (b) ENPCS-650 and (c) ENPCS-800 at 0.05 A g^{-1}.

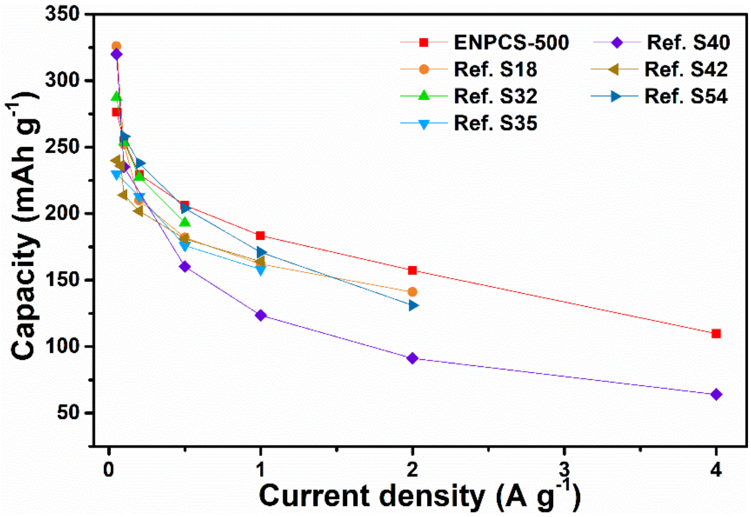

Figure S8. Comparison of rate performances of ENPCS-500 with some reported carbons.

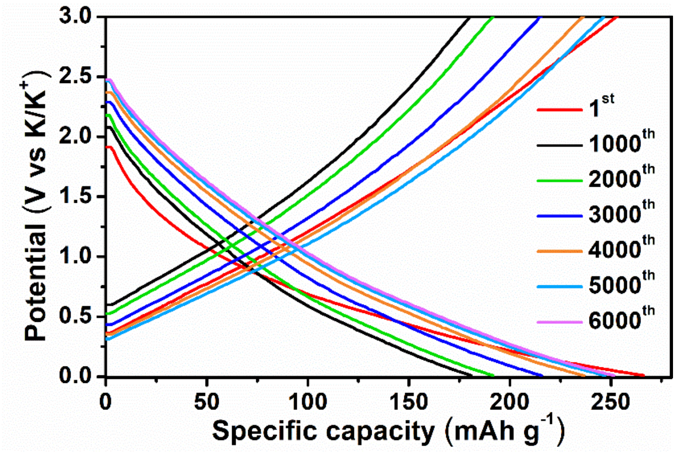

Figure S9. The galvanostatic discharge-charge profiles of ENPCS-500 at different cycles under the current density of 1 A g^{-1}. Note that the initial two discharge-charged cycles at 0.05 A g^{-1} were not included.

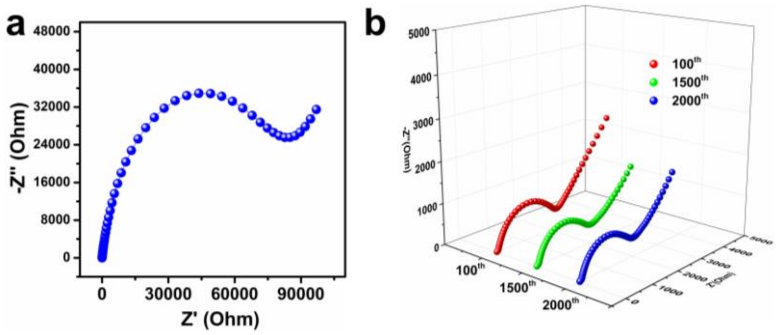

Figure S10. Electrochemical impedance spectra in Nyquist form for ENPCS-500 (a) before and (b) after different cycles at 1 A g^{-1}.

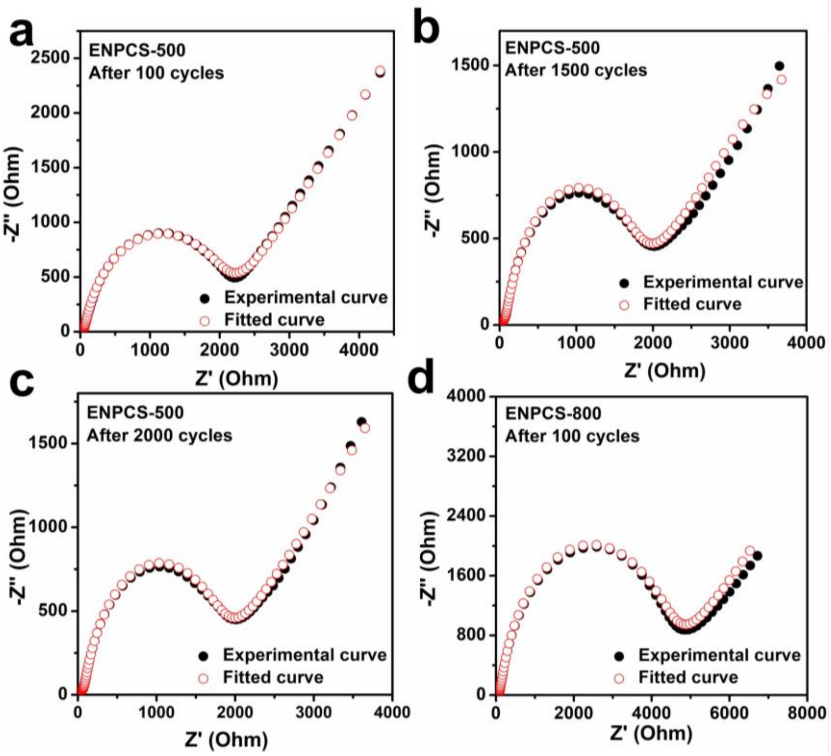

Figure S11. The experimentally measured plot and fitted electrochemical impedance spectra in the form of the Nyquist plot for (a-c) ENPCS-500 after different cycles and (d) ENPCS-800 after 100 cycles.

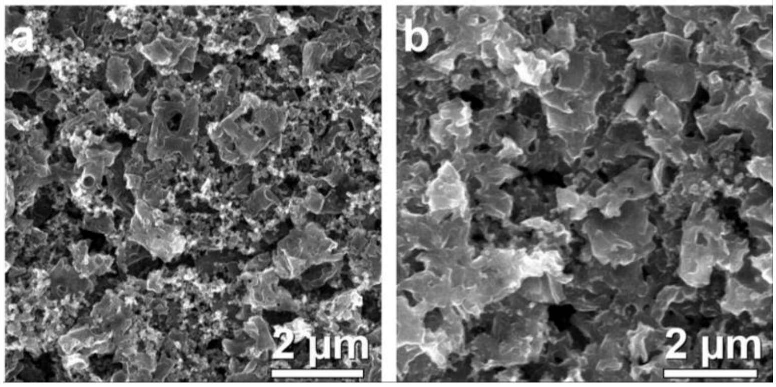

Figure S12. SEM images of ENPCS-500 electrode (a) before and (b) after 1000 cycles at 1 A g^{-1}.

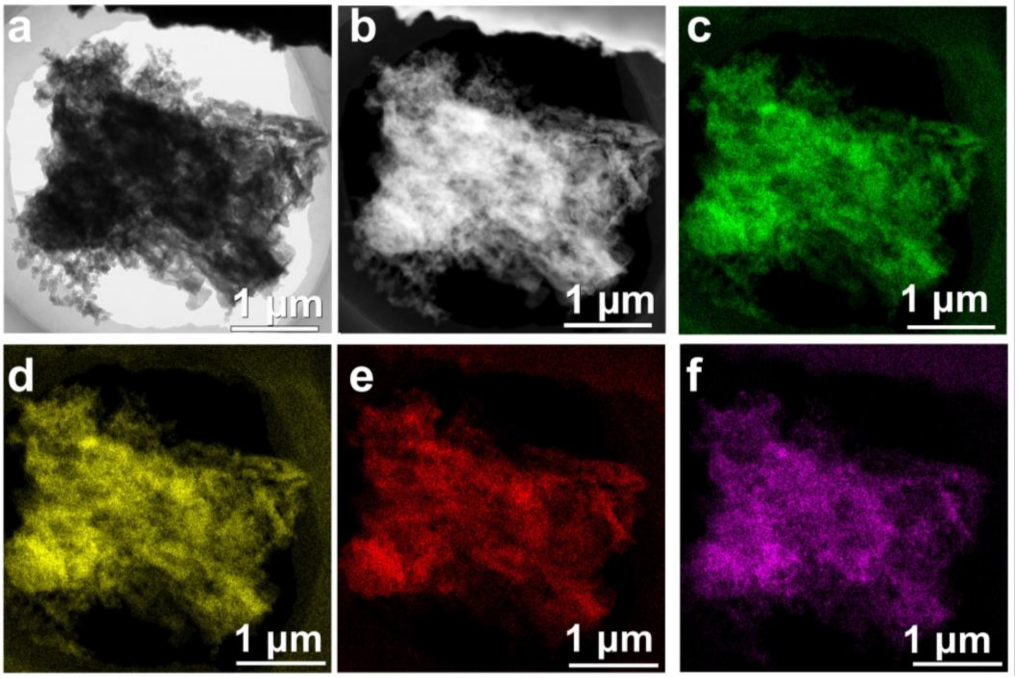

Figure S13. (a) TEM (b) HAADF-STEM images of ENPCS-500 electrode disassembled after 1000 cycles at 1 A g^{-1}, and the corresponding elemental mappings of (c) carbon, (d) nitrogen, (e) oxygen and (f) potassium.

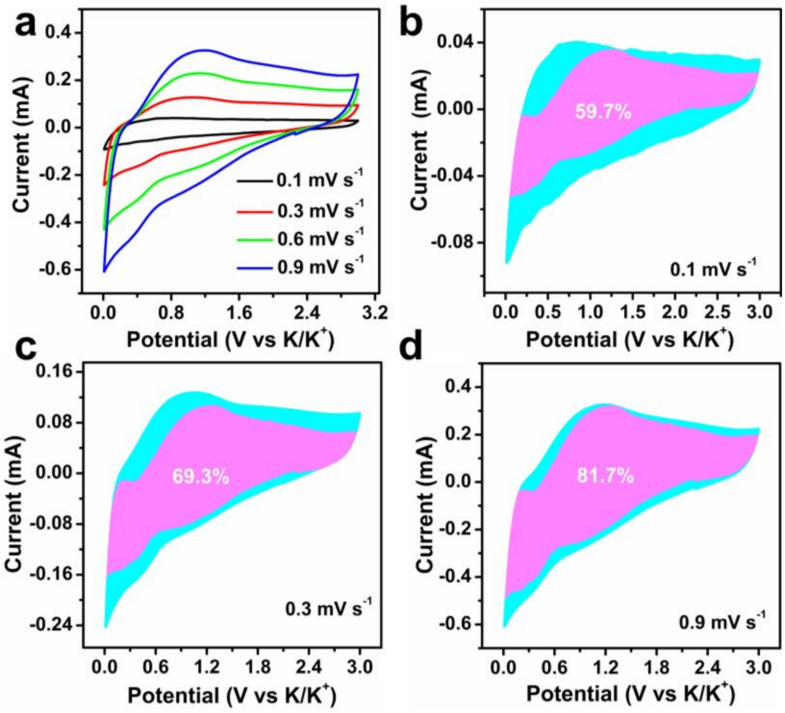

Figure S14. (a) CV curves at different scan rates of ENPCS-500, the separation of capacitive (pink region) and diffusion-controlled (cyan region) contribution at (b) 0.1 mV s^{-1}, (c) 0.3 mV s^{-1} and (d) 0.9 mV s^{-1} for ENPCS-500.

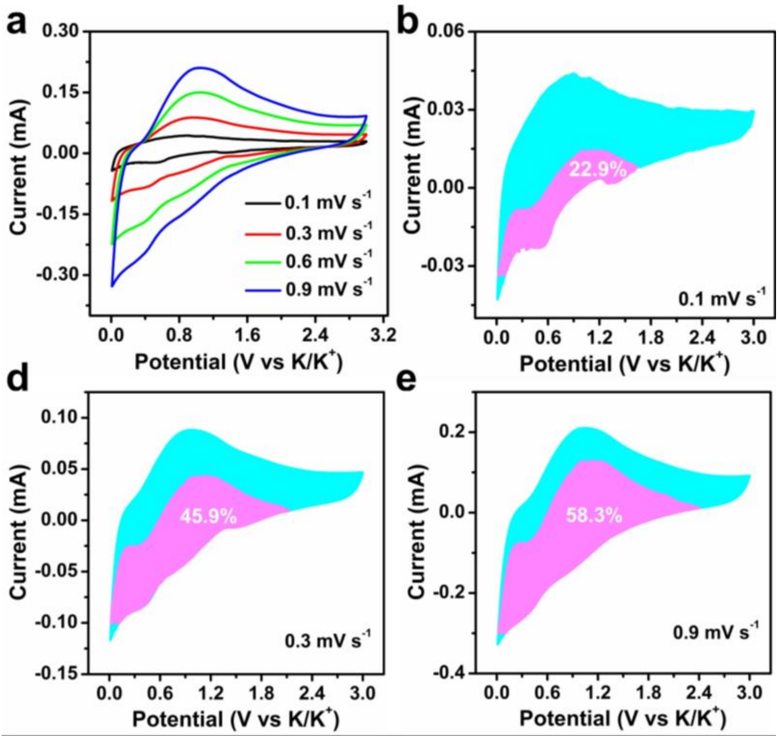

Figure S15. (a) CV curves at different scan rates of ENPCS-800, the separation of capacitive (pink region) and diffusion-controlled (cyan region) contribution at (b) 0.1 mV s^{-1}, (c) 0.3 mV s^{-1} and (d) 0.9 mV s^{-1} for ENPCS-800.

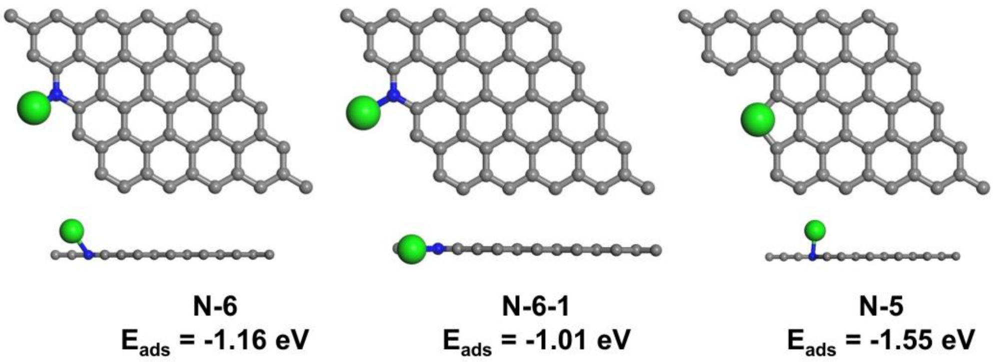

Figure S16. Relative adsorption energy (E_{ads}) of K atom on the edge plane of carbon rings for N-6 with two possible binding sites and N-5.

3. Supporting Tables

Table S1. Parameters determined from Raman spectra

Sample	Peak position (cm^{-1})		Peak area (%)		I_D/I_G
	D	G	D	G	
ENPCS-500	1358	1573	73.05	26.96	2.71
ENPCS-650	1357	1573	76.39	23.61	3.24
ENPCS-800	1356	1577	78.13	21.87	3.57

Table S2. Elemental composition determined by XPS and combustion analysis

Sample	XPS element content (at%)				Combustion element content (wt%)			
	C	N	O	N:C	C	N	H	N:C (at%)
ENPCS-500	80.42	11.76	7.82	1:6.8	61.14	15.30	1.57	1: 4.66
ENPCS-650	81.94	10.62	7.44	1:7.7	67.70	14.53	1.53	1: 5.44
ENPCS-800	86.38	7.42	6.20	1:11.6	72.47	8.95	1.32	1: 9.45

Table S3. Chemical configuration of N 1s and the corresponding content

Sample	Peak position (eV)			Peak area (%)		
	N-6	N-5	N-Q	N-6	N-5	N-Q
ENPCS-500	398.1	399.7	400.7	49.0	30.4	20.6
ENPCS-650	398.1	399.7	400.7	45.5	27.5	27.0
ENPCS-800	397.8	399.3	400.5	33.1	23.5	43.4

Table S4. Comparison of surface area and N content

Materials	BET surface area (m^2 g^{-1})	N content (at%)	Edge N content (at%)	Ref.
**ENPCS-500**	**616**	**11.76**	**9.34**	**This work**
Nitrogen/oxygen co-doped mesoporous carbon octahedrons	1411	1.09	1.09	[10]
Nitrogen/oxygen co-doped amorphous carbon	177	1.7	NG	[11]
N-doped graphene	NG	2.2	1.67	[12]
N-doped carbon nanotubes	126	2.4	2.4*	[13]
N/P dual-doped multilayer graphene	NG	3.2	2.57	[14]
N/P dual-doped vertical graphene	15	3.85	2.58	[15]
Nitrogen-doped hierarchical porous hollow carbon spheres	625	5.03	3.31	[16]
Nitrogen-doped graphitic nanocarbons (GNC600)	305	4.8	3.47	[17]
N-doped hollow carbon nanospheres	228	5.0	3.8	[18]
S/N dual-doped hard carbon	110	4.16	NG	[4]
Nitrogen-doped porous carbon	341	6.88	4.23	[19]
Nitrogen/ oxygen dual-doped hierarchical porous hard carbon	1030	7.06	4.77	[20]
N/O dual-doped carbon network	102	7.3	5.31	[21]
High pyridine N-doped porous carbon (NPC-600)	908	NG	5.32*	[22]
Ultrathin carbon nanosheets	828	5.8	NG	[23]
Chitin-derived N-doped carbon nanofibers	458	7.41	6.45	[24]
Highly N-doped carbon nanofibers (NCNF-650)	99	13.8	7.73	[25]
N/O-rich carbon nanofiber	153	7.8	NG	[26]
Carbon quantum dots tailored hollow carbon (SNCs)	18	9.03	8.00	[27]
Edge-nitrogen doped carbons	270	10.5	9.2	[28]
N-doped necklace-like hollow carbon	356	10.71	9.21	[29]
Ultrahigh pyridinic N-content-doped porous carbon monolith	443	18.9	10.1	[30]
Nitrogen-doped bamboo-like carbon nanotubes	150	12.12	11.24	[31]
Nitrogen-doped porous carbon	326	11.84	NG	[32]
Ultrahigh N-doped carbon nanosheets	54	22.7	17.34	[33]

***** represent only N-6 is provided

Table S5. Chemical state of O 1s and the corresponding content

Sample	Peak position (eV)			Peak area (%)		
	C=O	C-OH/C-O-C	COOH	C=O	C-OH/C-O-C	COOH
ENPCS-500	531.1	532.7	534.1	65.3	24.6	10.1
ENPCS-650	531.3	532.8	533.9	73.9	16.4	9.7
ENPCS-800	531.2	532.7	534.1	74.0	13.7	12.3

Table S6. Chemical state of C 1s and the corresponding content

Sample	Peak position (eV)				Peak area (%)			
	C=C/C-C	C-O/C-N	C=N	C=O	C=C/C-C	C-O/C-N	C=N	C=O
ENPCS-500	284.5	285.4	286.4	288.8	37.1	27.4	19.4	16.0
ENPCS-650	284.5	285.5	286.7	289.4	42.2	27.1	19.2	11.5
ENPCS-800	284.5	285.3	286.5	289.5	38.4	27.3	18.2	16.1

Table S7. The corresponding parameters from the equivalent circuit simulation

Sample	R_e (Ohm)	R_{ct} (Ohm)	R_x (Ohm)	CPE1	CPE2
ENPCS-500@100 cycle	13	1902	40	2.2E-05	1.2E-05
ENPCS-500@1500 cycle	12	1522	34	2.8E-05	9.2E-06
ENPCS-500@2000 cycle	13	1581	41	2.4E-05	6.5E-06
ENPCS-800@100 cycle	15	4061	52	2.3E-05	2.5E-06

Table S8. Cycling performance comparison of PIB anode materials

Sample		Current density (A g^{-1})	Cycle number	Capacity (mAh g^{-1})	Decay rate per cycle (%)	Retention ratio (%)	Reference
Heteroatom-doped carbons	PCC500	1.0	6000	251.5	0.0009	94.5	This work
	N/O dual-doped carbon network	1	4000	160	0.008	68	[21]
	N-doped carbon nanosheet	5	3000	110	0.01	70	[33]
	N-doped carbon spheres	1	3000	117.2	0.0066	80.1	[23]
	N-doped hollow carbons	1	2500	154	0.0156	61	[18]
	O/F dual-doped porous carbon	1	2000	218	0.004	92	[34]
	S/O co-doped porous carbon	1	2000	108.4	~0.01385	~72.3	[35]
	N-doped porous carbon	0.5	2000	231.6	~0.0139	~72.2	[22]
	N-doped carbon nanofibers	1	2000	164	~0.008	~84	[25]
		2	4000	146	~0.00225	~91	
	N/O-rich carbon nanofiber	0.279	1900	170	~0.0086	~83.5	[26]
	Necklace-like N-doped hollow carbon	1	1600	161.3	0.017	73	[29]
	N, O co-doped mesoporous carbon	1	1300	100	0.0185	76	[10]
	O-rich carbon nanosheets	2	1300	147	0.0139	82	[36]
	S/N dual-doped hard carbon	3	1200	144.9	0.0281	66	[4]
	N/O dual-doped hierarchical porous hard carbon	1.05	1100	124.8	0.028	69.5	[20]
	N/O co-doped amorphous carbon	1	1000	167	0.03	70	[11]
	N/O-codoped carbon hollow multihole bowls	1	1000	133	0.034	66	[37]
	N-doped carbon nanosheets	1	1000	151	0.0244	75.6	[38]
	P-doped hard carbon	0.2	1000	260	0.02	80	[39]
	N-doped Porous Carbon	1	1000	226.1	0.0117	88.3	[19]
	N-doped bamboo-like carbon nanotubes	0.5	1000	204	~0.0892	~10.8	[31]

	N-doped hollow carbon	1	800	160	0.02	84	[27]
	S, N co-doped thin carbon	2	900	65	-	-	[40]
	F-Doped graphene	0.5	200	165.9	0.25	51	[2]
	N-doped few-layered graphene	0.1	100	210	0.23	77	[12]
**Porous carbons**	Hierarchical carbon nanotubes	0.1	500	210	0.02	90	[41]
	Free-standing carbon nanofiber foam	1	2000	158	0.006	88	[42]
	Hollow interconnected carbon	0.28	500	~150	0.05	~75	[43]
	Nanonetwork-structured carbon	5	2000	108	0.004	92	[44]
	Yolk-shell carbon spheres	1	1200	138	0.005	94	[45]
	3D porous carbon nanofiber paper	0.2	1200	211	0.01	88	[46]
	Hard carbon spheres/soft carbon composite	0.28	200	200	0.035	93	[47]
	Mesoporous graphitic carbon	1	2000	223.6	0.009	81.7	[48]
	Ordered mesoporous carbon	1	1000	146.5	0.03	~70	[49]
	MoO_2/Reduced graphene oxide hollow sphere	0.5	500	104.2	0.08	60	[50]
	Phosphorus/carbon composite （PAC-35）	-	500	-	0.06	70	[51]
**Carbon-based composites**	VN-QDs/CM-600	0.5	500	215	0.02	90	[52]
	$MoSe_2$/N-doped carbon	2	500	-	~0.034	~83	[53]
	Bamboo-Like MoS_2/N-doped-C hollow tubes	0.5	1000	151	~0.026	~74	[54]
	Nb_2O_{5-x}@rGO	1.5	3500	81	~0.00286	~90	[55]
	V_2O_3@PNCNFs	0.05	500	~210	0.0084	95.8	[56]
	Ti_3C_2 MXene/N-rich porous carbon nanosheets	1	2000	252.2	0.03	40	[57]
	Ultrathin carbon film@ carbon nanorods@Bi	1	700	90	0.036	74.8	[58]

Table S9. Calculation of I_D/I_G via the in situ Raman spectra

Sample	Peak position (cm^{-1})		Peak area (%)		I_D/I_G
	D	G	D	G	
Discharge					
ENPCS-500-2.34 V	1353	1573	68.02	31.98	2.13
ENPCS-500-2.20 V	1356	1577	69.97	30.03	2.33
ENPCS-500-2.00 V	1352	1573	68.12	31.88	2.14
ENPCS-500-1.80 V	1354	1575	67.98	32.02	2.12
ENPCS-500-1.50 V	1356	1574	69.55	30.45	2.28
ENPCS-500-1.00 V	1356	1576	69.29	30.71	2.26
ENPCS-500-0.60 V	1353	1575	68.82	31.18	2.21
ENPCS-500-0.46 V	1357	1577	69.63	30.37	2.29
ENPCS-500-0.20 V	1355	1575	66.43	33.57	1.98
ENPCS-500-0.01 V	1359	1579	70.25	29.75	2.36
Charge					
ENPCS-500-0.50 V	1356	1575	69.95	30.05	2.33
ENPCS-500-1.00 V	1358	1578	69.66	30.34	2.30
ENPCS-500-1.50 V	1355	1574	67.88	32.12	2.11
ENPCS-500-1.80 V	1355	1574	69.18	30.82	2.24
ENPCS-500-2.00 V	1353	1575	68.35	31.65	2.16
ENPCS-500-2.50 V	1353	1573	68.81	31.19	2.21
ENPCS-500-3.00 V	1355	1574	69.25	30.75	2.25

4. Supporting References

[1] L. Zhang, Q. He, S. Huang, J. Zhu, J. Key, P. K. Shen, *Inorg. Chem. Commun.* **2018**, *96*, 159.
[2] Z. Ju, S. Zhang, Z. Xing, Q. Zhuang, Y. Qiang, Y. Qian, *ACS Appl. Mater. Inter.* **2016**, *8*, 20682.
[3] S. Yang, J. Huo, H. Song, X. Chen, *Electrochim. Acta* **2008**, *53*, 2238.
[4] Y. Liu, H. Dai, L. Wu, W. Zhou, L. He, W. Wang, W. Yan, Q. Huang, L. Fu, Y. Wu, *Adv. Energy. Mater.* **2019**, *9*, 1901379.
[5] G. Kresse, J. Furthmüller, *Comp. Mater. Sci.* **1996**, *6*, 15.
[6] G. Kresse, J. Furthmüller, *Phys. Rev. B* **1996**, *54*, 11169.
[7] J. P. Perdew, K. Burke, M. Ernzerhof, *Phys. Rev. Lett.* **1996**, *77*, 3865.
[8] G. Kresse, D. Joubert, *Phys. Rev. B* **1999**, *59*, 1758.
[9] P. E. Blöchl, *Phys. Rev. B* **1994**, *50*, 17953.
[10] G. Xia, C. Wang, P. Jiang, J. Lu, J. Diao, Q. Chen, *J. Mater. Chem. A* **2019**, *7*, 12317.
[11] Q. Sun, D. P. Li, J. Cheng, L. N. Dai, J. G. Guo, Z. Liang, L. J. Ci, *Carbon* **2019**, *155*, 601.
[12] K. Share, A. P. Cohn, R. Carter, B. Rogers, C. L. Pint, *ACS Nano* **2016**, *10*, 9738.
[13] X. X. Z. P. X. Xiong, Y.H. Xu, *ChemSusChem* **2018**, *11*, 202.
[14] Y. Luan, R. Hu, Y. Fang, K. Zhu, K. Cheng, J. Yan, K. Ye, G. Wang, D. Cao, *Nano-Micro Lett.* **2019**, *11*, 30.
[15] W. Qiu, H. Xiao, Y. Li, X. Lu, Y. Tong, *Small* **2019**, *15*, 1901285.
[16] D. Qiu, J. Guan, M. Li, C. Kang, J. Wei, Y. Li, Z. Xie, F. Wang, R. Yang, *Adv. Funct. Mater.* **2019**, *29*, 1903496.
[17] W. Zhang, J. Ming, W. Zhao, X. Dong, M. N. Hedhili, P. M. F. J. Costa, H. N. Alshareef, *Adv. Funct. Mater.* **2019**, *29*, 1903641.
[18] J. Ruan, X. Wu, Y. Wang, S. Zheng, D. Sun, Y. Song, M. Chen, *J. Mater. Chem. A* **2019**, *7*, 19305.
[19] D. Li, X. Ren, Q. Ai, Q. Sun, L. Zhu, Y. Liu, Z. Liang, R. Peng, P. Si, J. Lou, J. Feng, L. Ci, *Adv. Energy. Mater.* **2018**, *8*, 1802386.
[20] J. Yang, Z. Ju, Y. Jiang, Z. Xing, B. Xi, J. Feng, S. Xiong, *Adv. Mater.* **2018**, *30*, 1700104.

[21] J. Ruan, Y. Zhao, S. Luo, T. Yuan, J. Yang, D. Sun, S. Zheng, *Energy Storage Mater.* **2019**, *23*, 46.
[22] Y. Li, C. Yang, F. Zheng, X. Ou, Q. Pan, Y. Liu, G. Wang, *J. Mater. Chem. A* **2018**, *6*, 17959.
[23] J. Qin, H. M. Kheimeh Sari, C. He, X. Li, *J. Mater. Chem. A* **2019**, *7*, 3673.
[24] R. Hao, H. Lan, C. Kuang, H. Wang, L. Guo, *Carbon* **2018**, *128*, 224.
[25] Y. Xu, C. Zhang, M. Zhou, Q. Fu, C. Zhao, M. Wu, Y. Lei, *Nat. Commun.* **2018**, *9*, 1720.
[26] R. A. Adams, J. M. Syu, Y. P. Zhao, C. T. Lo, A. Varma, V. G. Pol, *Acs Appl. Mater. Inter.* **2017**, *9*, 17872.
[27] W. Hong, Y. Zhang, L. Yang, Y. Tian, P. Ge, J. Hu, W. Wei, G. Zou, H. Hou, X. Ji, *Nano Energy* **2019**, *65*, 104038.
[28] W. Zhang, Z. Cao, W. Wang, E. Alhajji, A. H. Emwas, P. Costa, L. Cavallo, H. N. Alshareef, *Angew. Chem. Int. Ed.* **2020**, *59*, 4448.
[29] W. Yang, J. Zhou, S. Wang, W. Zhang, Z. Wang, F. Lv, K. Wang, Q. Sun, S. Guo, *Energy Environ. Sci.* **2019**, *12*, 1605.
[30] Y. Xie, Y. Chen, L. Liu, P. Tao, M. Fan, N. Xu, X. Shen, C. Yan, *Adv. Mater.* **2017**, *29*, 1702268.
[31] Y. Liu, C. Yang, Q. Pan, Y. Li, G. Wang, X. Ou, F. Zheng, X. Xiong, M. Liu, Q. Zhang, *J. Mater. Chem. A* **2018**, *6*, 15162.
[32] X. Qi, K. Huang, X. Wu, W. Zhao, H. Wang, Q. Zhuang, Z. Ju, *Carbon* **2018**, *131*, 79.
[33] X. Chang, X. Zhou, X. Ou, C. S. Lee, J. Zhou, Y. Tang, *Adv. Energy. Mater.* **2019**, *9*, 1902672.
[34] J. Lu, C. Wang, H. Yu, S. Gong, G. Xia, P. Jiang, P. Xu, K. Yang, Q. Chen, *Adv. Funct. Mater.* **2019**, *29*, 1906126.
[35] M. Chen, W. Wang, X. Liang, S. Gong, J. Liu, Q. Wang, S. J. Guo, H. Yang, *Adv. Energy. Mater.* **2018**, *8*, 1800171.
[36] J. Chen, B. Yang, H. Hou, H. Li, L. Liu, L. Zhang, X. Yan, *Adv. Energy. Mater.* **2019**, *9*, 1803894.
[37] Z. Zhang, B. Jia, L. Liu, Y. Zhao, H. Wu, M. Qin, K. Han, W. A. Wang, K. Xi, L. Zhang, G. Qi, X. Qu, R. V. Kumar, *ACS Nano* **2019**, *13*, 11363.
[38] L. Liu, Y. Chen, Y. Xie, P. Tao, Q. Li, C. Yan, *Adv. Funct. Mater.* **2018**, *28*, 1801989.
[39] Y. Qian, S. Jiang, Y. Li, Z. Yi, J. Zhou, T. Li, Y. Han, Y. Wang, J. Tian, N. Lin, Y. Qian, *Adv. Energy. Mater.* **2019**, *9*, 1901676.
[40] A. Mahmood, S. Li, Z. Ali, H. Tabassum, B. Zhu, Z. Liang, W. Meng, W. Aftab, W. Guo, H. Zhang, M. Yousaf, S. Gao, R. Zou, Y. Zhao, *Adv. Mater.* **2019**, *31*, 1805430.
[41] Y. Wang, Z. Wang, Y. Chen, H. Zhang, M. Yousaf, H. Wu, M. Zou, A. Cao, R. P. S. Han, *Adv. Mater.* **2018**, *30*, 1802074.
[42] H. Li, Z. Cheng, Q. Zhang, A. Natan, Y. Yang, D. Cao, H. Zhu, *Nano Lett.* **2018**, *18*, 7407.
[43] D.-S. Bin, X.-J. Lin, Y.-G. Sun, Y.-S. Xu, K. Zhang, A.-M. Cao, L.-J. Wan, *J. Am. Chem. Soc.* **2018**, *140*, 7127.
[44] W. Zhang, Y. Yan, Z. Xie, Y. Yang, Y. Xiao, M. Zheng, H. Hu, H. Dong, Y. Liu, Y. Liang, *J. Colloid Interf. Sci* **2020**, *561*, 195.
[45] H. Zhang, H. He, J. Luan, X. Huang, Y. Tang, H. Wang, *J. Mater. Chem. A* **2018**, *6*, 23318.
[46] X. Zhao, P. Xiong, J. Meng, Y. Liang, J. Wang, Y. Xu, *J. Mater. Chem. A* **2017**, *5*, 19237.
[47] Z. Jian, S. Hwang, Z. Li, A. S. Hernandez, X. Wang, Z. Xing, D. Su, X. Ji, *Adv. Funct. Mater.* **2017**, *27*, 1700324.
[48] Y. Qian, S. Jiang, Y. Li, Z. Yi, J. Zhou, J. Tian, N. Lin, Y. Qian, *Angew. Chem. Int. Ed.* **2019**, *58*, 18108.
[49] W. Wang, J. H. Zhou, Z. P. Wang, L. Y. Zhao, P. H. Li, Y. Yang, C. Yang, H. X. Huang, S. J. Guo, *Adv. Energy. Mater.* **2018**, *8*, 1701648.
[50] C. Liu, S. Luo, H. Huang, Y. Zhai, Z. Wang, *ChemSusChem* **2019**, *12*, 873.
[51] X. Huang, D. Liu, X. Guo, X. Sui, D. Qu, J. Chen, *ACS Sustain. Chem. Eng.* **2018**, *6*, 16308.
[52] H. Wu, Q. Yu, C.-Y. Lao, M. Qin, W. Wang, Z. Liu, C. Man, L. Wang, B. Jia, X. Qu, *Energy Storage Mater.* **2019**, *18*, 43.
[53] J. Ge, L. Fan, J. Wang, Q. Zhang, Z. Liu, E. Zhang, Q. Liu, X. Yu, B. Lu, *Adv. Energy. Mater.* **2018**, *8*, 1801477.
[54] B. Jia, Q. Yu, Y. Zhao, M. Qin, W. Wang, Z. Liu, C.-Y. Lao, Y. Liu, H. Wu, Z. Zhang, X. Qu, *Adv. Funct. Mater.* **2018**, *28*, 1803409.
[55] Z. Tong, R. Yang, S. Wu, D. Shen, T. Jiao, K. Zhang, W. Zhang, C. S. Lee, *Small* **2019**, *15*, 1901272.
[56] T. Jin, H. Li, Y. Li, L. Jiao, J. Chen, *Nano Energy* **2018**, *50*, 462.
[57] R. Zhao, H. Di, X. Hui, D. Zhao, R. Wang, C. Wang, L. Yin, *Energy Environ. Sci.* **2020**, *13*, 246.
[58] S. Su, Q. Liu, J. Wang, L. Fan, R. Ma, S. Chen, X. Han, B. Lu, *Acs Appl. Mater. Inter.* **2019**, *11*, 22474.

Author Contributions

S.K. H.W. and F.X. conceived the concept and directed the research; F.X., Y.Z., E.Z. and Q.L. carried out the synthesis. Y.Z. performed the K-ion battery experiments. Y.Z. X.X. and Y.Q. performed structural characterizations. G.J. and X.L. gave advice to the research. F.X. and Y.Z. wrote the manuscript.
